# Where in 1902?

**Published:** 1901-12

**Authors:** 


### WHERE IN 1902?

Chicago and Kansas City, through their State organizations, are in the field for the meeting of the A. V. M. A. in 1902. Columbus and Cleveland have been considering the matter also ; while far-away Helena, Montana, coyly wishes that the Association may have courage sufficient to come out there. All of the places named have their respective attractions, and some need the stimulus and added strength of a national meeting in their midst; but the one great problem for the Executive Board to consider is what is best for the organization. Do we need to grow in numbers by additions of local members of the profession whose work has but a touch of national importance? Are we to take the field of State and local organizations in furnishing a post-graduate course of instruction, or are we to reach out and strengthen our organization among those who are working out great national and international problems that benefit a whole nation—in fact, the world of veterinarians? Is our national organization to be the field of exhibition for the adept veterinarian in performing neurectomies or firing spavins? Is it to be a field of instruction for the spaying of bitches, or ovariotomies of mares or cows under most adverse and unfortunate circumstances, or are we to endeavor each year to reach out and bring into the fold strong minds and broader advocates of the crying needs in higher veterinary education, greater sanitary control work, State legislation in its many aspects—the direction of State organizations along higher and better lines, and plans for representation on all State Live-stock Sanitary Boards, all State and local Health Boards, among all breeding organizations where laws and regulations are to be framed for increasing the wealth in this direction by safeguards best known to our profession, to encourage and foster our veterinary literature, strengthen and enhance our journalism, and in fact prove to be the clearing-house of distribution of all that goes to make up a truly national organization? Which way shall we lead?

---

WE recommend all of our readers and Association workers to specially note the Association proceedings of the Missouri State Veterinary organization. This active and progressive body has just enjoyed one of the most attractive meetings of the profession held by any State organization for several years. The activity of its officers in preparing so rich a programme for their meeting deserves much commendation, and it should be a guide and a stimulus to every other Association in the land. The social features in connection with their work added much to the delightful character of their meeting.

